# Synthesis and photodynamic antimicrobial chemotherapy against multi-drug resistant *Proteus mirabilis* of ornithine-porphyrin conjugates *in vitro* and *in vivo*

**DOI:** 10.3389/fmicb.2023.1196072

**Published:** 2023-06-08

**Authors:** Shuai Meng, Zengping Xu, Xueming Wang, Yang Liu, Bole Li, Jie Zhang, Xiaolong Zhang, Tianjun Liu

**Affiliations:** ^1^Department of Pharmacy, Tianjin Medical University Cancer Institute and Hospital, National Clinical Research Center for Cancer, Key Laboratory of Cancer Prevention and Therapy, Tianjin’s Clinical Research Center for Cancer, Tianjin, China; ^2^Tianjin Key Laboratory of Biomedical Materials, Institute of Biomedical Engineering, Chinese Academy of Medical Sciences and Peking Union Medical College, Tianjin, China; ^3^Center for Drug Evaluation, National Medical Products Administration, Beijing, China

**Keywords:** photodynamic antimicrobial chemotherapy, photosensitizer, cationic porphyrins, *Proteus mirabilis*, multi-drug resistant

## Abstract

For the treatment of bacterial infections, photodynamic antimicrobial chemotherapy (PACT) has the advantage of circumventing multi-drug resistance. In this work, new cationic photosensitizers against multi-drug resistant *Proteus mirabilis* (MRPM) were designed and synthesized by the conjugation of amino phenyl porphyrin with basic amino acid L-ornithine. Their photoinactivation efficacies against MRPM *in vitro* were reported and include the influence of laser energy, uptake, MIC and MBC, dose-dependent photoinactivation effects, membrane integrity, and fluorescence imaging. The PACT *in vivo* was evaluated using a wound mouse model infected by MRPM. Photosensitizer **4d** displayed high photo inactivation efficacy against MRPM at 7.81 μM under illumination, and it could accelerate wound healing via bactericidal effect. These ornithine-porphyrin conjugates are potential photosensitizers for PACT in the treatment of MRPM infection.

## 1. Introduction

*Proteus mirabilis* is a Gram-negative bacterial opportunistic pathogen that belongs to the *Morganellaceae* family of the *Enterobacterales* order. It can cause urethritis, bacteremia, pyelonephritis, otitis media, acute and chronic diarrhea in children, myelitis, diabetic foot infection, and other diseases (Schaffer and Pearson, 2015; Palusiak, 2022). In recent years, due to the intensive use of antibiotics, multi-drug resistant *P. mirabilis* (MRPM), which is non-susceptible to at least one agent in three or more antimicrobial categories (Magiorakos et al., 2012), has emerged in clinical treatment. Beta-lactam resistance was often found in MRPM due to the production of extended-spectrum beta-lactamases, AmpC enzymes, or metallo-beta-lactamases. Point mutations in *gyrA*, *gyrB,* and *parC* genes on chromosomes of *P. mirabilis* could cause resistance to quinolones (Dougnon et al., 2020; Santiago et al., 2020; Shaaban et al., 2022). Therefore, it is essential to discover efficient antimicrobial methods that apply different mechanisms for the treatment of *P. mirabilis* infection.

Photodynamic antimicrobial chemotherapy (PACT) is an alternative antibacterial method with a different mechanism from antibiotics, which uses photosensitizers and visible light to induce photodynamic inactivation against microbial pathogens (Oyim et al., 2021; Piksa et al., 2023). Irradiated by the appropriate wavelength of light, the photosensitizer undergoes an energy leap. The energy is transferred to oxygen in the organism to generate reactive oxygen species, which results in photodamage or the death of bacteria. PACT is mainly dependent on the oxidative damage to proteins, cell membranes, mitochondria, nuclei, or other cellular components of target cells by reactive oxygen species produced by photosensitizers, and thus it has the advantage of circumventing multi-drug resistance (Vickerman et al., 2021; Xiu et al., 2022).

In PACT, the photosensitizer is one of the key core factors. Since the positively charged groups can interact with the negatively charged lipopolysaccharides or peptidoglycans on the bacterial cell wall to improve the binding ability with bacteria, the photoinactivation ability of cationic porphyrin photosensitizers against gram-positive and gram-negative bacteria is usually stronger than that of neutral and negatively charged photosensitizers (Malatesti et al., 2017; Vieira et al., 2018; Li et al., 2020; Hu et al., 2022).

In our previous study, we designed and synthesized a series of amino acid-modified cationic photosensitizers. One of the compounds, **4i**, amino tetraphenyl porphyrin linking four L-lysine groups, could eradicate methicillin-resistant *Staphylococcus aureus* (MRSA), *E.coli*, and *P. aeruginosa* at 3.91 μM and 7.81 μM under illumination *in vitro* (Meng et al., 2015). It also exhibited promising treatment effects *in vivo* on a wound rat model infected by mixed bacteria including MRSA, *E. coli*, and *P. aeruginosa* (Xu et al., 2016). However, when it was used to treat clinically isolated MRPM, it displayed no activity (MIC >500 μM). Thus, we developed new photosensitizers.

In this work, new cationic photosensitizers against MRPM were developed and synthesized by the conjugation of amino tetraphenyl porphyrin with L-ornithine. L-ornithine belongs to basic amino acids, which makes the photosensitizers carry cationic charges at physiological pH value, thus promoting the affinity of microorganisms to the photosensitizers (Liu et al., 2022). In addition, L-ornithine is a component of the bacterial cell membrane and polypeptide antibiotics (Plapp and Kandler, 1967). Furthermore, the main physiological function of L-ornithine is to participate in the urea cycle which has a protective effect on the liver (Mohamed and Subramani, 2009; Harada et al., 2019). Those properties make porphyrin photosensitizers modified with L-ornithine gain good biocompatibility and high affinity to bacteria, which increases the uptake amount by MRPM and their photodynamic antibacterial activity. This paper reports the synthesis and their photoinactivation efficacies against MRPM *in vitro* including the influence of laser energy, uptake, MIC and MBC, dose-dependent photoinactivation effects, membrane integrity, fluorescence imaging, and the effectiveness *in vivo* on an MRPM-infected wound mouse model.

## 2. Materials and methods

### 2.1. General

Unless otherwise specified, all materials were used as received from commercial suppliers. The reagents were chemically or analytically pure. ^1^H and ^13^C NMR spectra were tested on a BRUCKER AVANCE 500 NMR spectrometer. Tetramethylsilane (TMS) is the internal standard. High resolution mass spectrometers (HRMS) were tested on Agilent 6,550 Q TOF and Thermo Fisher-QE. Compound **1** (5,10,15,20-tetrakis(4-nitrophenyl)porphyrin) and compound **2** (5,10,15,20-tetrakis(4-aminophenyl)porphyrin) were prepared as described in the literature (Semeikin et al., 1982).

### 2.2. Chemistry

The general procedure for the synthesis of compounds **4a–d** is as follows: A mixture of acid Boc-Orn(Boc)-OH (1.00 equiv), HATU (2.00 equiv), and *N, N*-Diisopropylethylamine (4.00 equiv) in *N,N*-Dimethylformamide (5 ml) was stirred at room temperature for 30 min. Then, 5,10,15,20-Tetrakis(4-aminophenyl)-*21H,23H*-porphine (0.125 equiv-0.40 equiv) was added to the acid mixture and the solution was stirred overnight at room temperature. The reaction was diluted with water (10 ml) and extracted with dichloromethane (3 × 10 ml). The combined organic layers were washed with brine, dried with anhydrous sodium sulfate, and concentrated. Purification with column chromatography on silica gel (200–300 mesh) gave the compounds **3a–d** (yield 47–80%). Trifluoroacetic acid (TFA/CH_2_Cl_2_, 1/1, v/v) was added to the solution of compounds **3a–d** (1 equiv) in dried CH_2_Cl_2_. The reaction was stirred for 30 min at room temperature and concentrated. Into the residue was added ethyl ether (10 ml) and a green solid was obtained by filtration. The solid was suspended in water (5 ml) and neutralized with ammonium hydroxide. The purple solid (**4a–d**) was obtained by filtration and washed with water (3 × 5 ml) and CH_2_Cl_2_ (3 × 5 ml; yield 54–83% for the two steps). The NMR and HRMS spectra of compounds **4a–d** are available in the Supplementary material.

#### 2.2.1. 5-(4-((S)-2,5-diaminopentanamido)phenyl)-10,15,20-tris(4-aminophenyl)porphyrin **(4a)**

95.3 mg, 54% yield; mp > 300°C. ^1^H NMR (300 MHz, DMSO-*d*_6_) δ8.78–8.88 (m, 8H), 8.22–8.00 (m, 4H), 7.83 (d, *J* = 7.9 Hz, 6H), 6.98 (d, *J* = 7.9 Hz, 6H), 5.57 (s, 6H), 3.45 (s, 1H), 2.71–2.53 (m, 2H), 1.78–1.48 (m, 4H), −2.76 (s, 2H). ^13^C NMR (75 MHz, DMSO*-d_6_*) δ 175.06, 148.86, 148.80, 139.18, 136.63, 135.99, 135.84, 131.79, 129.17, 129.11, 129.03, 121.76, 121.37, 121.12, 119.24, 118.11, 113.28, 113.00, 57.07, 42.60, 33.96, 30.98. IR (KBr): *v* 3294.67, 3098.75, 3029.90, 2921.86, 2853.22, 1673.42, 1608.31, 1517.87, 1471.93, 1350.42, 1286.23, 1278.78, 1178.45, 966.32, 820.31, 803.12, 731.21 cm^−1^. HRMS (ESI-TOF): *m/z* calcd for C_49_H_44_N_10_O 811.3592 [M + Na]^+^, found 811.3585.

#### 2.2.2. 5,10-di(4-((S)-2,5-diaminopentanamido)phenyl)-15,20-di(4-aminophenyl)porphyrin **(4b)**

95.3 mg, 77% yield; mp > 300°C. ^1^H NMR (300 MHz, DMSO-*d*_6_) δ 8.90–8.80 (m, 8H), 8.13–8.00 (s, 8H), 7.84 (d, *J* = 7.8 Hz, 4H), 6.98 (d, *J* = 7.8 Hz, 4H), 5.58 (s, 4H), 3.45 (t, *J* = 6.1 Hz, 2H), 2.61 (s, 4H), 1.77–1.47 (m, 8H), −2.80 (s, 2H). ^13^C NMR (75 MHz, DMSO-*d*_6_) δ 175.26, 149.10, 139.44, 136.71, 136.14, 135.29, 131.77, 129.18, 122.20, 119.69, 118.34, 113.45, 113.22, 57.24, 42.71, 36.58, 34.01. IR (KBr): *v* 3286.85, 3098.29, 3029.89, 2922.15, 2853.51, 1670.43, 1607.21, 1518.46, 1470.64, 1349.75, 1308.05, 1278.65, 1178.37, 966.05, 823.56, 799.31, 731.34 cm^−1^. HRMS (ESI-TOF): *m/z* calcd for C_54_H_54_N_12_O_2_ 903.4565 [M + H]^+^, found 903.4554.

#### 2.2.3. 5,10,15-tris(4-((S)-2,5-diaminopentanamido)phenyl)-20-(4-aminophenyl)porphyrin **(4c)**

95.3 mg, 61% yield; mp > 300°C. ^1^H NMR (300 MHz, DMSO-*d*_6_) δ 8.83–8.91 (m, 8H), 8.11 (s, 14H), 7.96–7.79 (m, 2H), 3.47 (t, *J* = 6.0 Hz, 3H), 2.81–2.57 (m, 6H), 1.94–1.40 (m, 12H), −2.87 (s, 2H). ^13^C NMR (75 MHz, DMSO*-d_6_*) δ 175.10, 154.10, 139.37, 136.35, 135.04, 131.74, 120.32, 118.45, 118.17, 56.74, 42.75, 34.09, 30.86. IR (KBr): *v* 3283.99, 3097.39, 3029.69, 2922.51, 2855.49, 1672.55, 1598.14, 1519.12, 1471.11, 1401.37, 1310.69, 1238.78, 1180.89, 966.42, 850.86, 799.23, 731.92 cm^−1^. HRMS (ESI-TOF): *m/z* calcd for C_59_H_64_N_14_O_3_ 1017.5359 [M + H]^+^, found 1017.5364.

#### 2.2.4. 5,10,15,20-trakis(4-((S)-2,5-diaminopentanamido)phenyl)porphyrin **(4d)**

95.3 mg, 83% yield; mp > 300°C. ^1^H NMR (300 MHz, DMSO-*d*_6_) δ 8.84 (s, 8H), 8.08–8.14 (m, 16H), 3.46 (d, *J* = 6.9 Hz, 4H), 2.76–2.55 (m, 8H), 1.90–1.43 (m, 16H), −2.90 (s, 2H). ^13^C NMR (75 MHz, DMSO*-d_6_*) δ 175.14, 139.37, 136.33, 135.11, 131.86, 120.35, 118.17, 56.93, 42.87, 34.13, 30.91. IR (KBr): *v* 3282.90, 3029.79, 2923.94, 2856.56, 1671.23, 1597.65, 1518.97, 1470.75, 1400.80, 1309.56, 1247.10, 1180.81, 1110.91, 966.54, 851.49, 798.53, 731.87 cm^−1^. HRMS (ESI-TOF): *m/z* calcd for C_64_H_74_N_16_O_4_ 1131.6152 [M + H]^+^, found 1131.6134.

### 2.3. Photosensitizers and light source

The photosensitizers were dissolved in dimethyl sulfoxide or phosphate buffered saline (PBS) to get the stock solution (500 mM). Light of 650 nm was outputted via an optic fiber from a semiconductor laser (WSLS-650/532-500 m-200 M-H2, Wavespectrum, China). The transmitted energy was calibrated with an optical power meter (LM1; Carl Zeiss). The irradiance of 3.33 mW/cm^2^ and doses of 0, 2, 4, 6, 8, 10, and 12 J/cm^2^ were used in the *in vitro* experiments while 100 mW/cm^2^ and 50 J/cm^2^ were used *in vivo.*

### 2.4. Bacterial culture

The MRPM used in the study was isolated from diabetic foot wounds of clinical patients at the First Teaching Hospital of Tianjin University of Traditional Chinese Medicine in 2016. The bacterial strain was inoculated in Luria Bertani (LB) liquid medium (10 ml) and cultured in a shaker at 37°C until the logarithmic growth phase. Then the bacterial solution was inoculated on LB solid medium and incubated overnight at 37°C. A single colony was selected and inoculated in a 5 ml liquid medium and cultured overnight (37°C, 220 rpm). After centrifugation, the concentration of bacteria was adjusted to 1 × 10^8^ CFU/ml and stored at 4°C.

We determined the antimicrobial susceptibility profile of the MRPM strain by following the methods in Clinical and Laboratory Standards Institute documents M07 and M100 (CLSI M07-A10 and M100-ED32). The broth macrodilution method was used to obtain the minimal inhibitory concentration (MIC) of antibiotics. The antimicrobial agents were diluted to different concentrations with Mueller-Hinton broth culture medium in aseptic test tubes, and then the bacteria were added to the test tube with a final concentration of 10^5^ CFU/ml. After inoculation, the test tube was placed in an incubator at 37°C for 24 h. The minimal concentration of the antimicrobial agents making bacterial suspension visibly change from turbid to clear was the MIC.

### 2.5. Uptake experiments

The standard curves were prepared as follows: The photosensitizers were dissolved in 0.1 M-1% SDS. The final concentrations were 31.25, 15.62, 7.81, 3.91, 1.95, 0.98, 0.49, and 0.24 μM. Then, 100 μl of the solution was added to a 96-well culture plate and four replicate wells were prepared for each concentration. The fluorescence intensity of each well was measured at an excitation wavelength of 418 nm and an emission wavelength of 658 nm. The standard curve was plotted according to the fluorescence intensity and concentrations.

Next, 1 ml of bacterial suspension in the logarithmic growth phase was centrifuged (9,000 rpm, 2 min) and then the supernatant was decanted, washed once with PBS, and resuspended in PBS to the corresponding optical density (OD_600_ = 0.6–0.8). Then, 10 μM of each compound, which was dissolved in PBS, was added and incubated for 0 min, 10 min, 20 min, 30 min, 40 min, 80 min, 160 min, and 320 min in the dark. The suspensions were centrifuged (9,000 rpm, 2 min) and washed with PBS. The bacteria were treated with 1% SDS for 24 h. The fluorescence intensity was measured. The amount of photosensitizer uptake by bacteria was calculated according to the standard curve. The change of the uptake amount along varying concentrations (0–31.25 μM) was measured using the same method, only the incubation time was 40 min. Three sets of independent experiments were performed.

### 2.6. The effects of irradiation energy density on PACT

Measures of 100 μl of MRPM suspension (OD_600_ = 0.1) and 100 μl of the photosensitizers (the final concentration = 15.62 μM) were loaded into a 96-well plate. Plates were kept in the dark for 40 min and then illuminated with a dose of light (0, 2, 4, 6, 8, 10, and 12 J/cm^2^) and a wavelength of 650 nm. After illumination, 100 μl of the aliquots were taken from each well and pipetted into a tube containing 900 μl of sterilized distilled water to make a 10^−1^ dilution. In addition, 10^−2^, 10^−3^, 10^−4^, 10^−5^, and 10^−6^ dilutions were prepared using the same method. Each dilution (100 μl) was loaded onto LB agar plates for culture. The colony-forming units (CFU) were statistically analyzed and the bacterial survival fractions were calculated according to the ratios of CFU of the administration groups and control group. Three sets of independent experiments were performed.

### 2.7. MIC and MBC determination

In a series of test tubes, MRPM suspension (500 μl) and photosensitizers (500 μl) were added. The final concentrations of photosensitizers and MRPM were 1.96, 3.91, 7.81, 15.62, 31.25, 62.50, 125, 250, and 500 μM and 10^6^ CFU/ml. The mixture was incubated in a shaker (180 rpm) for 40 min in the dark and then irradiated by laser (650 nm, 8 J/cm^2^). After being cultured in the dark for 24 h, the degree of turbidity of the bacterial solution was observed. Then, 100 μl samples from the tubes without turbidity were added to LB agar plates and incubated at 37°C for 24 h. The minimum bactericidal concentration (MBC) was determined when no more than five colonies were observed on the plate. Three sets of independent experiments were performed.

### 2.8. Dose-dependent photoinactivation effects

The bacteria (OD_600_ = 0.1) and photosensitizers (0, 1.95, 3.91, 7.81, 15.62, and 31.25 μM) were incubated in the dark at 37°C for 40 min. The suspensions were then illuminated (650 nm, 8 J/cm^2^). The remaining procedure is the same as that detailed in the section “The effects of irradiation energy density on PACT.” Three sets of independent experiments were performed.

### 2.9. Membrane integrity

#### 2.9.1. Fluorescence imaging

Bacterial membrane damage was considered to be a sign of cell death. The membrane integrity of the bacteria was detected by an inverted fluorescence microscope (Nikon Eclipse Ti/B0004, Nikon, Japan) using the acridine orange/ethidium bromide (AO/EB) double fluorescence staining method. Photosensitizers **4b**, **4c**, and **4d** (15.62 μM) and bacterial suspensions (OD_600_ = 0.1) were incubated in a 96-well plate for 40 min at 37°C in the dark. The suspensions were illuminated with 8 J/cm^2^ (650 nm) light. After irradiation, the suspensions were centrifuged (9,000 rpm, 2 min) and washed with 0.9% NaCl (1 ml) and resuspended. Then, 4 μl of 100 mg/L AO and 100 mg/L EB mixed double fluorescent dyes were added and incubated for 5 min in the dark. After the bacteria were stained, their fluorescence was observed with an inverted fluorescence microscope at 200× magnification.

#### 2.9.2. Exudation study of bacterial contents

Leakage of cytoplasmic contents including ions, DNA, RNA, and other substances is a characteristic indication of bacterial membrane damage. The change of absorbance at 260 nm can be used to estimate the amount of contents leaked from bacteria (Sahu et al., 2009). The experiment was conducted by comparing the absorbance of four groups for each photosensitizer: (A) PBS treatment group; (B) light alone (8 J/cm^2^); (C) photosensitizer alone (PS alone); and (D) photosensitizer + PACT (8 J/cm^2^) (PS-PACT). After treatment, the bacterial solution of each group was filtered with a 0.22 μm filter membrane to remove bacteria and the OD_260_ absorbance of samples was recorded.

### 2.10. Excisional wound model and establishment of infection

The wound healing effect was evaluated using BALB/C mice. The animal experimental protocols were all approved by the Animal Ethical and Welfare Committee of Tianjin Medical University Cancer Institute and Hospital. The weight of BALB/C mice from Beijing HFK Bioscience Co., Ltd. was 20–22 g. Mice in each group were anesthetized via intraperitoneal injection of 10% chloral hydrate (300 mg/kg). After local skin preparation on the back, the skin was incised in its entirety to the deep fascia and a 1.0 cm diameter circular incision was created. Then the suspension (50 μl) of MRPM (2 × 10^9^ CFU/ml) in PBS was inoculated onto the surface of the wound and then smeared with an inoculating loop. Next, gauze was used to bandage the wound. After 48 h, when the wound infection model was successful, it was used in the next experiment.

### 2.11. 4d-PACT *in vivo*

Four groups including 24 mice with wound infections were involved in the experiments *in vivo*: (A) no treatment control; (B) light alone (50 J/cm^2^); (C) 4d-PACT; and (D) 4d alone (without illumination). The mice in groups C and D were treated with 50 μl of 4d (100 μM in 0.9% NaCl solution) which was injected into the wound. After 40 min in the dark, the mice wounds of groups B and C were irradiated with the light (650 nm, 50 J/cm^2^) while the other areas of the mice were shielded from light. The next day, the mice were irradiated again at the same dose of light without photosensitizer administration. The above process was defined as one PACT treatment. The PACT treatment was performed three times.

The day the mice were first treated with photosensitizers was considered day 1. On days 1, 2, 3, 4, 5, 6, 7, 8, 10, and 12, the wound area was calculated by measuring the length and width. The rate of wound healing of the mice was obtained by the following formula. Wound healing ratio = unhealed wound area/initial wound area. The viability of mice after treatment was observed and recorded. Meanwhile, the number of bacterial colonies in the wound at different time points was counted according to the following methods. A sterile cotton swab was dipped into normal saline and evenly smeared the whole wound from one side to the other side, and immediately put into an aseptic tube. During the detection, the ratio of normal saline was diluted to 10^−6^, and then 100 μl of the bacterial solution was loaded onto LB agar plates for culture. The colony morphology was observed and counted.

### 2.12. Statistical analysis

Data were analyzed by using SPSS 19.0 and were expressed as mean ± standard error of mean (SEM) or mean ± standard deviation (SD). Significant differences between sample means were determined by least significant difference *t*-tests or *χ*^2^ test.

## 3. Results

### 3.1. Synthesis of the ornithine-porphyrin conjugates

**Scheme 1:**
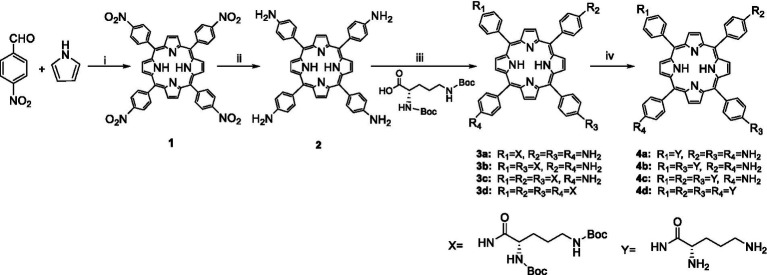
Synthesis of ornithine-porphyrin conjugates **4a–d**. Reagents and conditions: (i) Ac_2_O/CH_3_CH_2_COOH, reflux, 0.5 h; (ii) SnCl_2_·2H_2_O/HCl, 80°C, 4 h; (iii) HATU, DIPEA, rt., overnight; and (iv) TFA, DCM, rt., 0.5 h.

New cationic photosensitizers against MRPM were designed and synthesized by the condensation of 5,10,15,20-tetrakis(4-aminophenyl)porphyrin (**2**) with Boc-protected ornithine as depicted in Scheme 1. The 5,10,15,20-tetrakis(4-aminophenyl)porphyrin (**2**) was prepared according to the method in the literature (Semeikin et al., 1982), and **3a–d** were synthesized by condensation of 5,10,15,20-tetrakis(4-aminophenyl)porphyrin with Boc-Orn(Boc)-OH catalyzed by HATU/DIPEA at room temperature. By adjusting the ratio of reactants, ornithine-porphyrin conjugates containing one to four ornithine side chains were successfully synthesized. Then, by using trifluoroacetic acid (TFA), the *t*-butyloxy carbonyl group was removed to get photosensitizers **4a–d**.

### 3.2. Effect of laser energy dose on PACT

**Figure 1:**
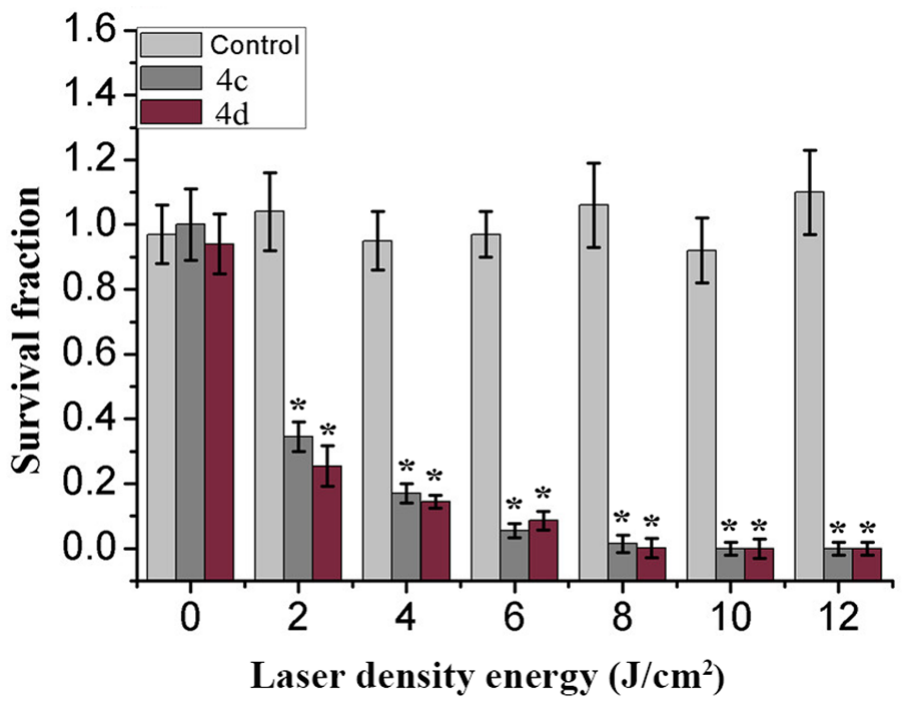
Effect of control (Light gray), 4c (Gray), or 4d (Purple) on MRPM strain as a factor of laser energy dose illumination at 650 nm, *p < 0.05 versus control group. The results are expressed as mean and standard deviation of three independent assays.

Energy dose is a main parameter essential for the therapeutic effect of photosensitizers. We evaluated the effect of laser energy density on photoinactivation abilities. MRPM suspensions containing compound **4c** or **4d** and the control group were irradiated with different energy densities. Laser irradiation alone had no significant inactivation efficiency against MRPM in the control group. The killing effect of photosensitizers increased with the increase of laser energy density, and it tended to be stable when the energy reached 8 J/cm^2^ (Figure 1). Moreover, based on our previous study on porphyrin derivatives (Meng et al., 2015; Xu et al., 2016; Rong et al., 2021; Yin et al., 2022), the best wavelength for treatment was 650 nm, which has strong penetration in tissue. Therefore, the condition of 650 nm, 8 J/cm^2^ was chosen for the subsequent experiments.

### 3.3. Uptake of ornithine-porphyrin conjugates by MRPM

**Figure 2:**
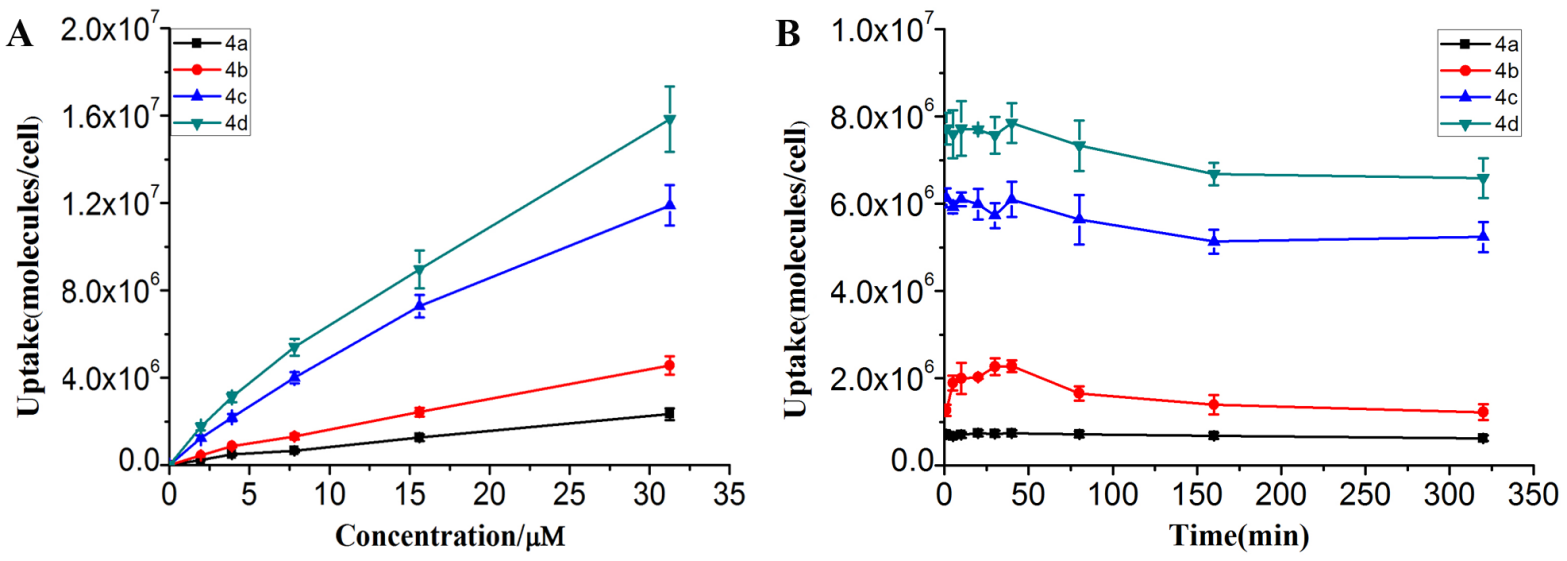
The uptake condition of MRPM toward ornithine-porphyrin conjugates. **(A)** The uptake of MRPM toward different concentration (0–31.25 μM) of ornithine-porphyrin conjugates. **(B)** The uptake of MRPM toward ornithine-porphyrin conjugates at different time points (0–320 min). The results are expressed as mean and standard deviation of three independent assays.

The amount of photosensitizer entering the bacteria has an important influence on the PACT efficacy. Therefore, the uptake experiment was performed using the fluorescence spectrophotometry method (Figure 2). Figure 2A shows that the uptake amount of photosensitizers by MRPM increased with the increasing concentration. Photosensitizer **4d**, linking four ornithine groups in porphyrin, showed the highest affinity for MRPM. It took about 40 min when the uptake amount of photosensitizers by MRPM reached the maximum and then entered the plateau period (Figure 2B). Therefore, in the following evaluation of antibacterial activity, 40 min was selected as the time point of incubation, that is, bacteria and photosensitizers were incubated for 40 min in the dark and then irradiated.

### 3.4. MIC and MBC determinations

The MIC and MBC of ornithine-porphyrin photosensitizers were determined to evaluate the photodynamic antibacterial activity against MRPM preliminary. The MRPM used in this experiment was isolated from the clinic. The antimicrobial susceptibility profile of the MRPM strain showed that it was resistant to a variety of antibiotics (Table 1). However, the MIC and MBC experiments demonstrated that photosensitizers **4c** and **4d** exhibited an inhibitory effect at concentrations of 15.62 μM and 7.81 μM under laser irradiation conditions (Table 2) when their dark toxicities were relatively weak (MIC ≥500 μM).

**Table 1:** Antimicrobial susceptibility profile of the MRPM strain.

Antibacterial drugs	MIC (mg/L)	Susceptibility profile
Aztreonam	32	R
Ampicillin	≥32	R
Tazobactam sodium/piperacillin sodium	≤4	S
Cefazolin	≥64	R
Ceftriaxone sodium	≥64	R
Imipenem	2	S
Gentamicin	≥16	R
Tobramycin	≥16	R
Amikacin	≤2	S
Cotrimoxazole	≥320	R
Ciprofloxacin	≥4	R
Levofloxacin	4	I
Furantoin	128	R
Tigecycline	4	I

S, susceptible; R, resistant; I, intermediate.

**Table 2:** MIC and MBC of ornithine-porphyrin conjugates against MRPM with or without illumination.

Compounds	MIC (μM)		MBC (μM)	
	8 J/cm^2^	0 J/cm^2^	8 J/cm^2^	0 J/cm^2^
4a	>500.00	>500.00	>500.00	>500.00
4b	>500.00	>500.00	>500.00	>500.00
4c	15.62	>500.00	31.25	>500.00
4d	7.81	500.00	15.62	>500.00

### 3.5. Dose-dependent photoinactivation effects

**Figure 3:**
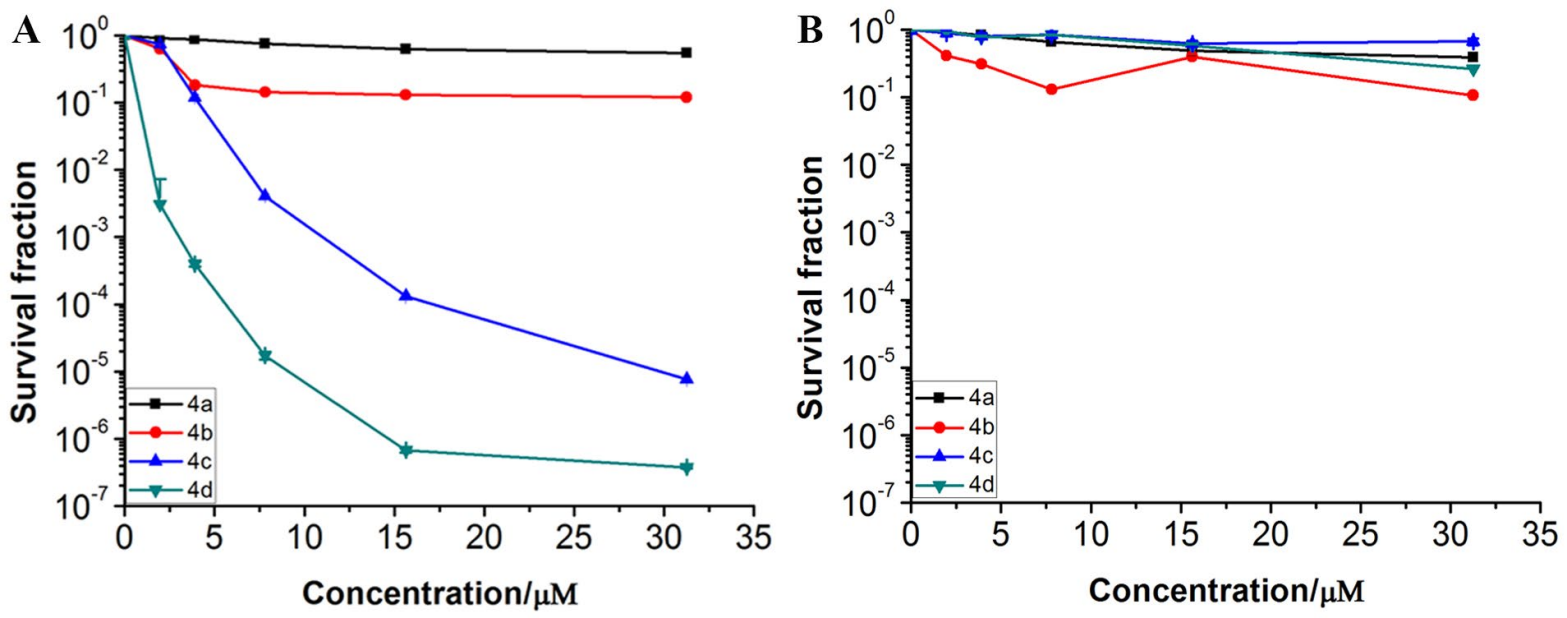
Photoinactivation effects **(A)** and dark toxicity **(B)** of ornithine-porphyrin conjugates against MRPM. The bacteria was incubated with compounds (0–31.25 μM) for 40 min, followed by irradiation (650 nm, 8 J/cm^2^) or darkness. The results are expressed as mean and standard deviation of three independent assays.

To further investigate the photodynamic bactericidal activities against MRPM of the ornithine-porphyrin photosensitizers, the dose-dependent photoinactivation effects were evaluated using the screened conditions. Generally, the MRPM suspension was incubated with the photosensitizers in the dark at 37°C for 40 min. The final concentrations of the photosensitizers included 0, 1.95, 3.91, 7.81, 15.62, and 31.25 μM and the irradiation energy was 8 J/cm^2^. Figure 3 shows the photoinactivation effects and dark toxicity against MRPM differed among the photosensitizers. The photodynamic inactivation effect against bacteria by photosensitizers was significantly dose-dependent, which increased with the increase of concentration (Figure 3A). When the dark toxicity of all compounds was very low, this indicated the photosensitizers had no bactericidal effect without irradiation (Figure 3B).

### 3.6. Membrane integrity

The cationic photosensitizers killed gram-negative bacteria more efficiently than neutral and negatively charged analogs by damaging the membrane, which is due to the ionic interaction between the cationic groups on photosensitizers and the negatively charged lipopolysaccharides or peptidoglycans on the bacterial cell wall. Therefore, the membrane integrity of ornithine-porphyrin conjugates was investigated by fluorescence microscopic imaging and measuring the release of intracellular components. Since compounds **4a** and **4b** both have weak activities, one of the compounds, **4b**, was chosen as a representative to be investigated.

**Figure 4:**
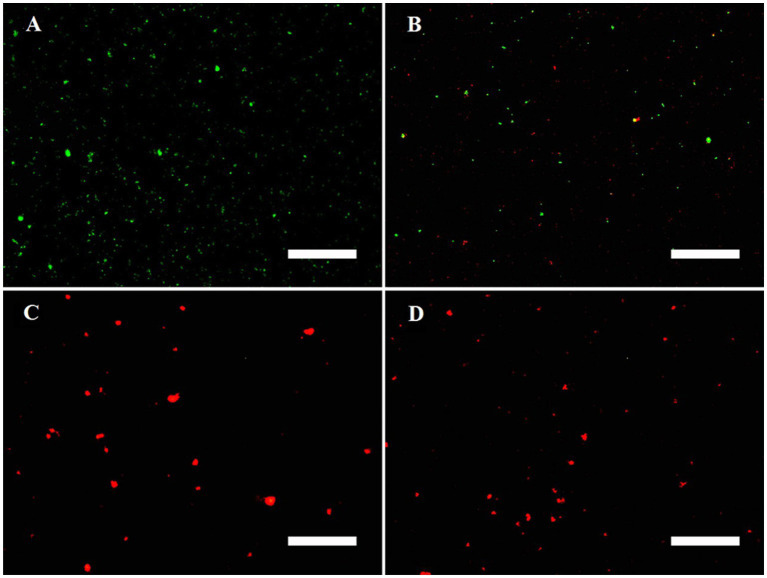
Fluorescence microscopy imaging of MRPM in control **(A)**, 4b-PACT **(B)**, 4c-PACT **(C)**, and 4d-PACT **(D)** groups. Bar represents 50 μm.

Figure 4 shows the fluorescence microscopy imaging of MRPM including the control (A), 4b-PACT (B), 4c-PACT (C), and 4d-PACT (D) groups. Acridine orange (AO) can penetrate the intact cell membrane and intercalate into DNA to make it emit bright green fluorescence. Ethidium bromide (EB) can only penetrate the damaged cell membrane, embed into DNA, and emit orange-red fluorescence. The fluorescence image of the control group exhibited dispersed green fluorescent particles, indicating the bacteria were alive and the membrane was not damaged. The 4b-PACT group had green and little red fluorescent particles, indicating the cell membrane of a small number of bacteria was ruptured. Most of the bacteria of 4c-PACT and 4d-PACT were dead with a red color due to the damage to the membrane. The results showed that the photosensitizers could destroy cell membranes to kill bacteria and the PACT effect of **4c** and **4d** was stronger than that of **4b**, which may be related to their physical and chemical properties and affinity to bacteria.

In addition, the destruction of bacteria membrane integrity could result in intracellular components including small ions and large molecules being released from bacteria. Large molecules such as DNA and RNA have strong UV absorption at the wavelength of 260 nm. Hence, the change of absorbance at the wavelength of 260 nm can reveal the integrity of the bacterial membrane (Sahu et al., 2009). After treatment with PS-PACT, the bacterial content exudation of the **4c** group and **4d** group were increased by 116.65, and 356.67%, respectively, while other groups including the PBS treatment group, light alone (8 J/cm^2^), and photosensitizer alone group changed from 2.01 to 9.31% (Table 3). These results demonstrated that 4c-PACT and 4d-PACT could cause disruption of bacterial cell membranes and changes in the permeability of MRPM.

**Table 3:** The increase of MRPM exudation in the control (PBS), PS alone, light alone (8 J/cm^2^), and PS-PACT (8 J/cm^2^) treatment groups (*n* = 4, mean ± SD) measured at 260 nm.

Group	Increase in OD_260_ relative to pre-treatment (%)			
	Control	PS alone	Light alone	PS-PACT
4b group	2.01 ± 0.23	6.31 ± 0.18	4.76 ± 0.55	11.53 ± 0.46
4c group	3.98 ± 0.33	7.34 ± 0.35	5.06 ± 0.68	116.65 ± 20.06^*^
4d group	2.98 ± 0.33	9.31 ± 0.38	5.76 ± 0.65	356.67 ± 28.08^*^

* *p* < 0.05 versus control group.

### 3.7. *In vivo* antibacterial activity and wound-healing effect

**Figure 5:**
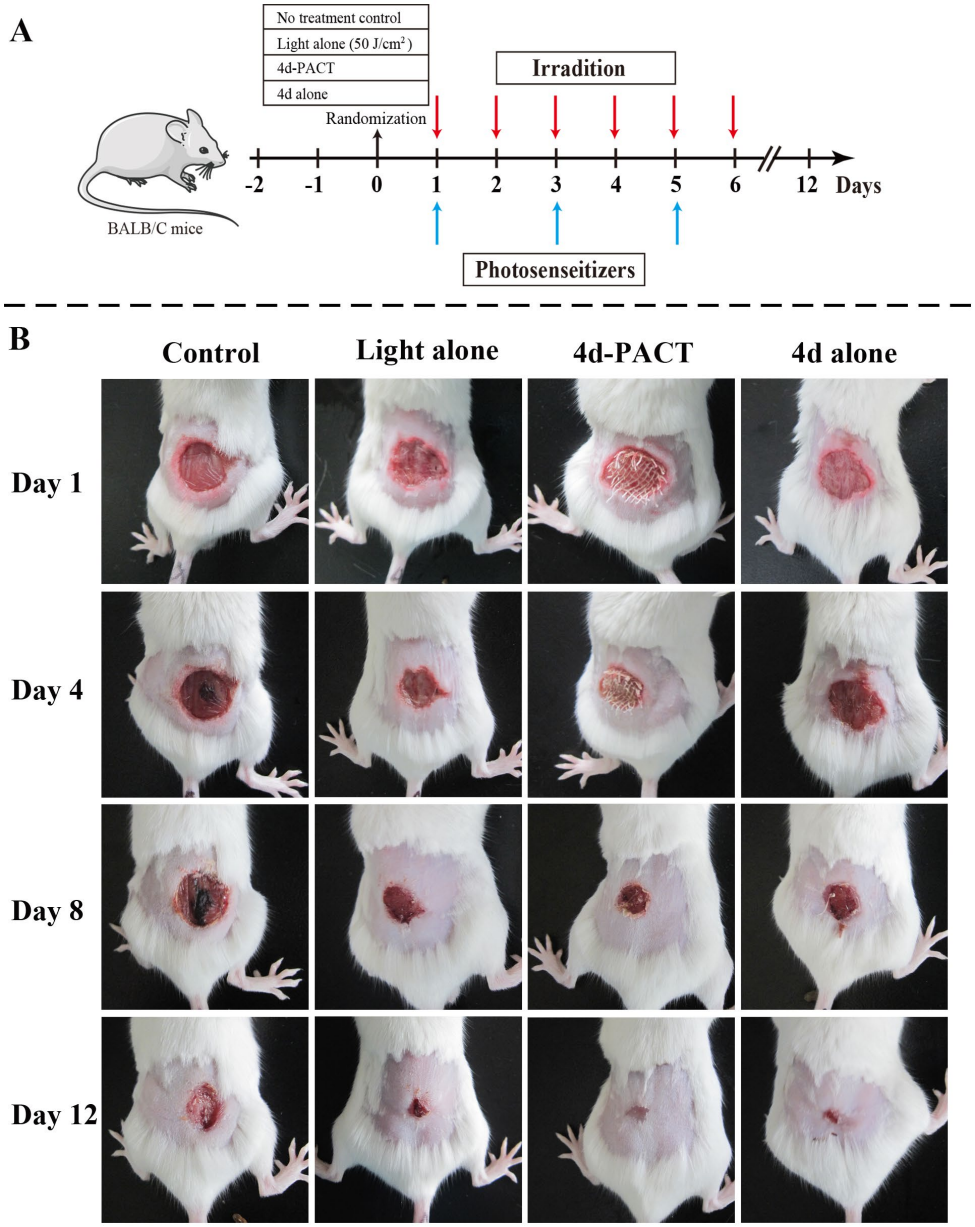
The antibacterial activity and wound-healing effect of 4d *in vivo*. **(A)** The diagrammatic representation of the 4d-PACT treatment *in vivo*; **(B)** The wound-healing effect of each group on day 1, 4, 8, and 12. The groups included the no-treatment control, light alone (50 J/cm^2^), 4d-PACT, and 4d alone (without illumination) groups.

**Figure 6:**
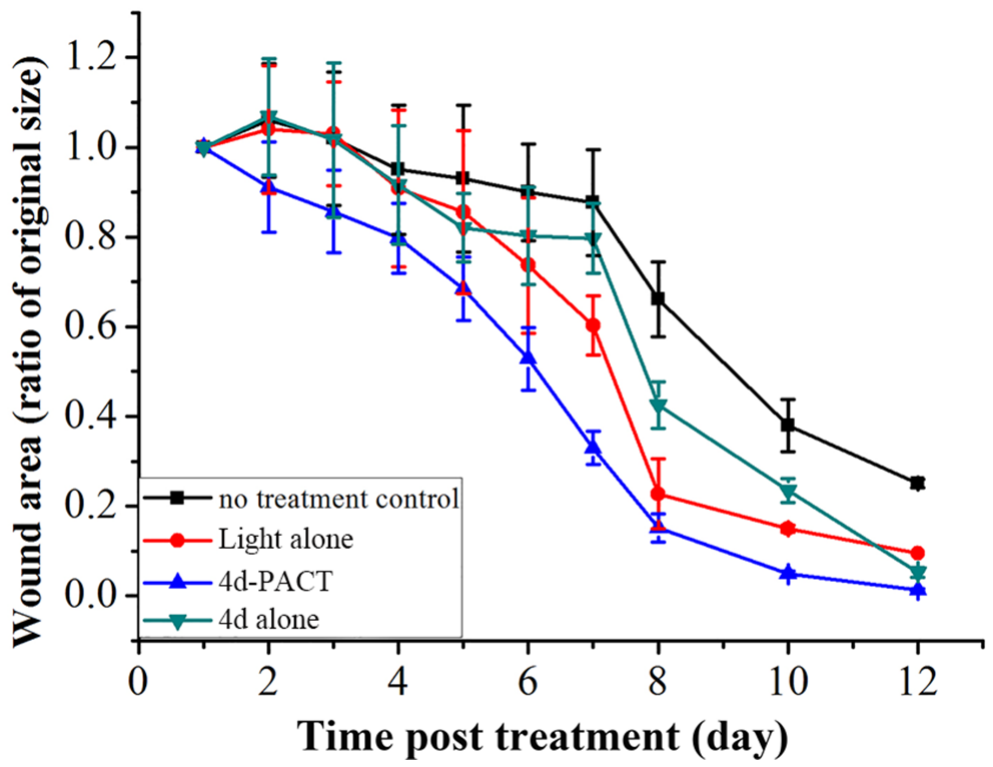
The change of wound area over time in MRPM-infected mice. The groups included the no-treatment control, light alone (50 J/cm^2^), 4d-PACT, and 4d alone (without illumination) groups. Each point represents the mean ratio of the area of the original wound. Data is expressed as mean ± SEM. *N* = 6 in each group.

The antibacterial effect of 4d-PACT *in vivo* was evaluated using a MRPM-infected wound mouse model. After the establishment of the model, the mice were randomly divided into four groups: no treatment control; light alone (50 J/cm^2^); 4d-PACT; and 4d alone (without illumination). The mice in the 4d-PACT group were treated with photosensitizers and irradiated. On the next day, the mice were irradiated again at the same dose of light without photosensitizer administration. The day the mice were first treated was considered day 1 (Figure 5A). The results showed that 4d-PACT was effective in the treatment of wounds infected by MRPM (Figure 5B). Compared with the control group with no treatment, the mice of the 4d-PACT treatment group showed rapid wound healing, especially between days 3 and 8 (*p* < 0.05). In the light alone (50 J/cm^2^) and 4d alone groups, rapid wound healing happened from day 5 to 8 and day 7 to 10, respectively, which was later than that of the 4d-PACT treatment group (Figure 6). The mean wound area ratios on day 8 of the control group, 4d-PACT treatment group, light alone group, and dark toxicity group were 0.68 ± 0.08, 0.16 ± 0.02, 0.23 ± 0.07, and 0.42 ± 0.05, respectively. The wound healed almost completely on day 12, although wound healing slowed down after day 8. These results indicated photosensitizer **4d** had better photodynamic efficiency on MRPM-infected wound healing.

**Figure 7:**
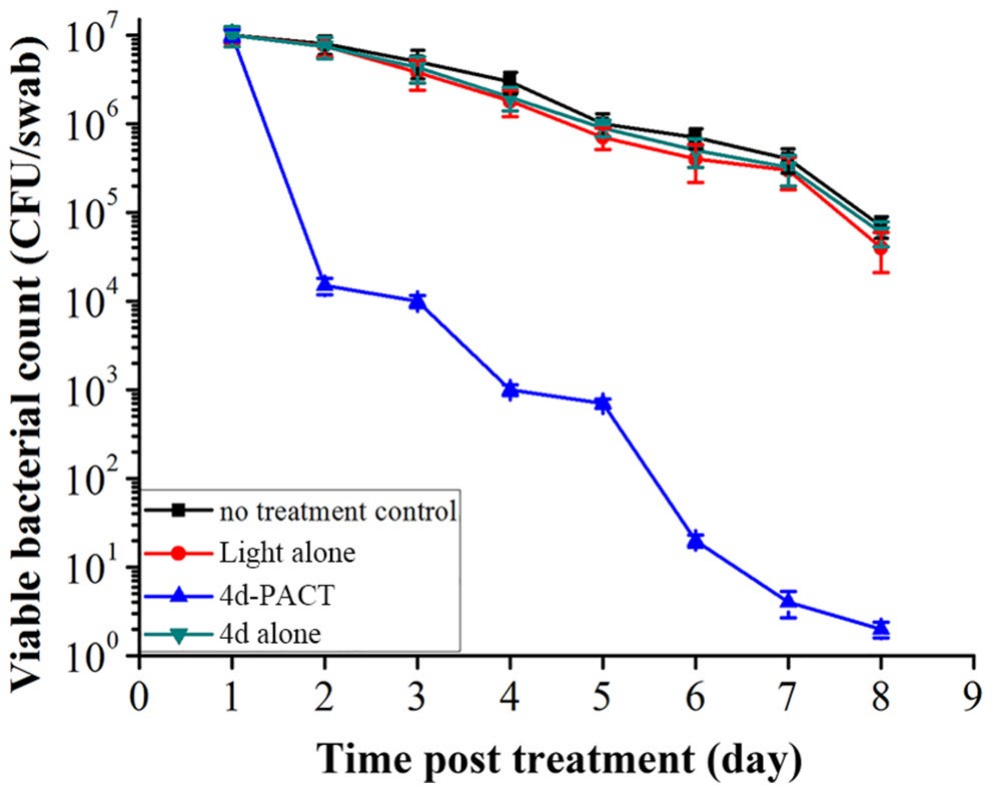
The viability of bacteria in the infected mice wounds at different time points in each treatment group post treatment. The groups included the no-treatment control, light alone (50 J/cm^2^), 4d-PACT, and 4d alone (without illumination) groups.

The bactericidal effect to MRPM of 4d-PACT was evaluated by counting the viable bacteria in wound tissue on days 1, 2, 3, 4, 5, 6, 7, and 8 (Figure 7). Compared with the other three groups, the 4d-PACT treatment group showed a significant decrease in the number of bacteria. However, there was little decrease in bacteria in the dark toxicity group and 50 J/cm^2^ laser irradiation group, which indicated that 4d-PACT accelerated wound healing by inhibiting the proliferation of MRPM.

**Figure 8:**
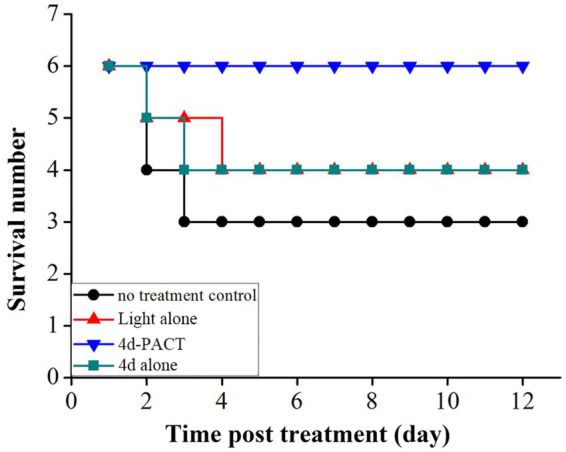
The survival time curve of mice in each experimental group. The groups included the no-treatment control, light alone (50 J/cm^2^), 4d-PACT, and 4d alone (without illumination) groups.

**Figure 9:**
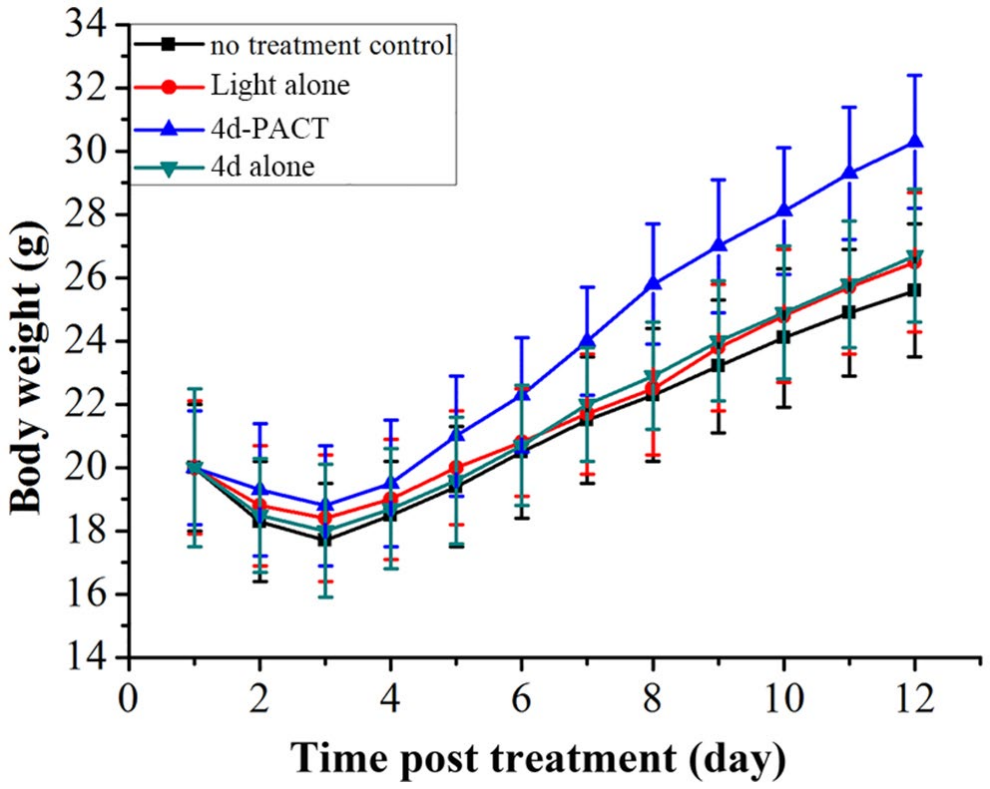
Body-weight change of MRPM-infected wound mouse models in each treatment group. The groups included the no-treatment control, light alone (50 J/cm^2^), 4d-PACT, and 4d alone (without illumination) groups.

Two mice died on day 2 and one died on day 3 in the control group, while a total of two mice died in the light alone (50 J/cm^2^) group, and a total of two mice also died in the 4d alone (dark toxicity) group. The mice in the 4d-PACT group all survived during the experiment, which indicated that 4d-PACT was more efficient in the treatment of MRPM infection (Figure 8). The change in the body weight of the mice is shown in Figure 9. On the whole, the mice had varying degrees of weight gain over 12 days, while there was a small decrease in body weight in the first 2 days after the establishment of the wound bacterial infection model. Compared with the control group, the mice in the 4d-PACT treatment group lost less weight in the first 2 days and gained more rapidly in the later stage. This showed that the body recovery of mice treated with 4d-PACT was faster than that of the other three groups, which was consistent with the results of wound healing rate and wound colony count.

## 4. Discussion

With the intensive use of antibiotics, MRPM emerged and its clinical isolation rate increased year by year. MRPM has become an important conditional pathogen, which exists in large numbers in intestinal and hospital environments. It can cause primary and secondary infections, such as in the urinary system, wounds, and respiratory tract, as well as other infections. *P. mirabilis* is naturally resistant to colistin and tigacycline and can form a biofilm, which greatly reduces the sensitivity of bacteria to antimicrobial agents. Furthermore, it can evade the host’s immune system and is difficult to destroy, which brings great difficulties to clinical anti-infection treatment (Kang et al., 2021; Yang et al., 2023).

Photodynamic antimicrobial chemotherapy, as one alternative therapeutic modality, uses light and photosensitizers to produce reactive oxygen species (ROS) by electron transfer (type I reaction) or energy transfer (type II reaction). These reactive oxygen species interact with many bacterial components to cause oxidative damage, resulting in bacteria death. It is not easy for bacteria to develop resistance to PACT (Marasini et al., 2021; Mohamad et al., 2023). The outer membrane of gram-negative bacteria is rich in negatively charged lipopolysaccharide molecules, which is naturally attractive to positively charged photosensitizers. It has been demonstrated that porphyrins bearing cationic groups can induce the photoinactivation of gram-positive and gram-negative bacteria and are usually more efficient than neutral and negatively charged analogs to bacteria (Malatesti et al., 2017). However, there are few reports of photosensitizers for the treatment of MRPM (Clerici et al., 2023; Elagin et al., 2023).

In this study, cationic photosensitizers against MRPM were developed and synthesized by the conjugation of amino tetraphenyl porphyrin with the basic amino acid L-ornithine. MRPM was clinically isolated and showed resistance to a variety of antibiotics such as Aztreonam, Ceftriaxone sodium, Tobramycin, Furantoin, Gentamicin, Cotrimoxazole, Ampicillin, Cefazolin, and Ciprofloxacin. After screening conditions including optimal irradiation dose and incubation time were determined, the PACT efficacy of those photosensitizers *in vitro* and *in vivo* was studied.

The MIC/MBC and dose-dependent photoinactivation effects experiments demonstrated that photosensitizer **4c** and **4d** showed good photoinactivation effects and eradicated MRPM at a rate of 3-log_10_ and 5-log_10_ at the concentration of 10 μM. It seems that the number of ornithine side chains affected the photodynamic bactericidal activities of photosensitizer and the activities increased with the increase of ornithine number. The order of photodynamic bactericidal activities was **4d** > **4c** > **4b** > **4a**. Photosensitizer **4d**, linking four ornithine side chains, exhibited the highest photodynamic bactericidal activity among those compounds. It could eradicate MRPM at a reduction rate of 3-log_10_ at 2.5 μM, while it needed 10 μM for photosensitizer **4c**. Photosensitizer **4b** and **4a**, with porphyrins bearing two and one ornithine moieties respectively, showed weak effects. This may be related to the amount of charge carried by the photosensitizer and the uptake amount by bacteria.

In order to further prove that photosensitizers could destroy the structure of bacteria and kill bacteria, the membrane integrity of MRPM treated with PACT was investigated by fluorescence microscopic imaging and measuring the release of intracellular components. The results confirmed that photosensitizers could destroy cell membranes and change the permeability of MRPM, resulting in the death of bacteria. The other phenomenon was also found in the fluorescence image. After PACT treatment, the bacteria with red fluorescence were agglomerated, while the bacteria with green fluorescence were uniformly dispersed. The reason for this was that the living MRPM could be uniformly dispersed because they could move, but after photodynamic treatment of **4c** and **4d**, most of the MRPM were killed, deposited, and agglomerated, and fluorescent staining presented in clusters.

The efficacy *in vivo* of ornithine-porphyrin conjugates was comprehensively evaluated including the wound-healing rate, the number of bacterial colonies under the wound at different times, and the survival rate of mice in each group by using the wound infection mouse model. The experimental results showed that the indices of the 4d-PACT treatment group were better than those of the control groups. The wound of the 4d-PACT treatment group healed faster than other groups and no mice died during the treatment. Moreover, the bacteria in the wound were effectively killed by 4d-PACT, which eradicated MRPM completely after the eighth day. Those therapeutic effects can be attributed to the dual effects that PACT not only killed bacteria but also led to the damage and repair of normal tissues (Mai et al., 2017).

These ornithine-porphyrin conjugates exhibited good photodynamic antibacterial activities against MRPM. Those can be attributed to the introduction of the basic amino acid L-ornithine that make the photosensitizers positively charged under physiological conditions, enhancing the interaction with the anionic groups on the bacterial cell wall and increasing the affinity with bacteria. On the other hand, the introduction of amino acids improves the water solubility of tetraphenyl porphyrin, and the amino acids, as a component of the cell wall, make them more readily absorbed by bacteria.

## 5. Conclusion

Since the previous photosensitizers had no activity against multi-drug resistant MRPM, we designed and synthesized new cationic porphyrin conjugates by condensation of 5,10,15,20-tetrakis(4-aminophenyl)porphyrin with Boc-protected ornithine and deprotection. Their photoinactivation efficacies against MRPM *in vitro* including the influence of laser energy, uptake, MIC and MBC, dose-dependent photoinactivation effects, membrane integrity, and fluorescence imaging were studied. Also, the PACT *in vivo* was evaluated using a wound mouse model infected with MRPM. Photosensitizer **4d** with a porphyrin bearing four ornithine moieties exhibited high photoinactivation ability. MRPM could be effectively eradicated at a concentration of 7.81 μM under illumination (8 J/cm^2^, 650 nm). Fluorescence microscopy imaging and absorbance at 260 nm confirmed that 4d-PACT can damage bacterial membrane integrity and alter their permeability. The wound healing effect on wound mouse models infected by MRPM demonstrated that 4d-PACT accelerated wound healing *via* bactericidal effect against MRPM *in vivo* and promoted normal tissue repair. Furthermore, 4d-PACT was also effective in increasing the survival of mice. These results demonstrate that the potential application of ornithine-porphyrin conjugates as cationic photosensitizers against MRPM is promising in the emerging field of PACT.

## Data availability statement

The original contributions presented in the study are included in the article/Supplementary material, further inquiries can be directed to the corresponding author.

## Ethics statement

The animal study was reviewed and approved by the Animal Ethical and Welfare Committee of Tianjin Medical University Cancer Institute and Hospital.

## Author contributions

TL and SM contributed to resources and conceptualization. SM, ZX and TL performed data curation and writing– review and editing. SM, XW, BL, XZ and YL performed formal analysis. TL and JZ performed investigation and supervision. XW, BL, ZX, JZ and XZ contributed to the methodology. SM, ZX, BL, JZ, XZ and TL performed validation. All authors contributed to the article and approved the submitted version.

## Funding

This work was supported by the CAMS Innovation Fund for Medical Sciences (2021-I2M-1-015/2021-I2M-1-052), Tianjin Municipal Science and Technology Project (grant 20JCYBJC00110), Tianjin Municipal Education Commission Foundation (grant 2019KJ187), Tianjin Key Medical Discipline (Specialty) Construction Project (TJYXZDXK-009A), Natural Science Foundation of China (grant 82000197) and Tianjin Medical University Cancer Hospital “14th Five-Year Plan” Summit Discipline Support Project – Outstanding Potential Discipline (7-2-11).

## Conflict of interest

The authors declare that the research was conducted in the absence of any commercial or financial relationships that could be construed as a potential conflict of interest.

## Publisher’s note

All claims expressed in this article are solely those of the authors and do not necessarily represent those of their affiliated organizations, or those of the publisher, the editors and the reviewers. Any product that may be evaluated in this article, or claim that may be made by its manufacturer, is not guaranteed or endorsed by the publisher.
