# Erratum: Synthesis and photodynamic antimicrobial chemotherapy against multi-drug resistant *Proteus mirabilis* of ornithine-porphyrin conjugates *in vitro* and *in vivo*

**DOI:** 10.3389/fmicb.2023.1260368

**Published:** 2023-08-09

**Authors:** Frontiers Production Office

**Affiliations:** Frontiers Media SA, Lausanne, Switzerland

**Keywords:** photodynamic antimicrobial chemotherapy, photosensitizer, cationic porphyrins, *Proteus mirabilis*, multi-drug resistant

Due to a production error, the captions did not match the figures correctly.

The artwork of Scheme 1 was missed and replaced by Figure 1, causing all subsequent figures to bear the caption of the next figure.

The publisher apologizes for this mistake. The original article has been updated.

