# Supplementary material for: Synthesis and photodynamic antimicrobial chemotherapy against multi-drug resistant Proteus mirabilis of ornithine-porphyrin conjugates in vitro and in vivo
Source: Front Microbiol. 2023 Jun 8;14:1196072. doi: 10.3389/fmicb.2023.1196072 (PMC10285166; doi:10.3389/fmicb.2023.1196072)
Supplement: Supplementary file 1 [file Data_Sheet_1.docx]

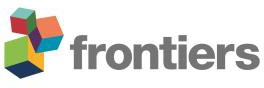


***Supplementary Material***

Synthesis and Photodynamic Antimicrobial Chemotherapy against Multi-drug Resistant Proteus mirabilis of Ornithine-Porphyrin Conjugates *in Vitro* and *in Vivo*

1 Supplementary Figures

Supplementary Figures 1 to 12 were NMR and HRMS spectra of compounds 4a-d.

Supplementary Material


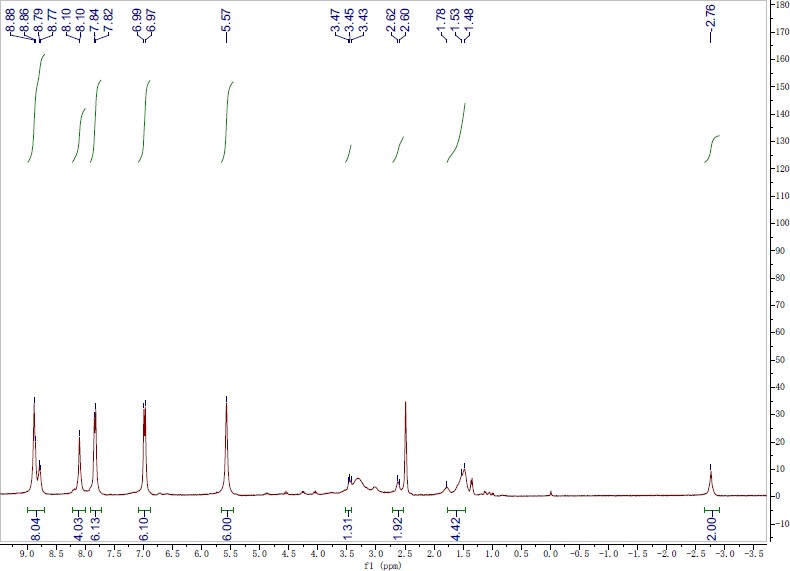


**Supplementary Figure 1.** ^1^H-NMR spectrum of compound **4a**


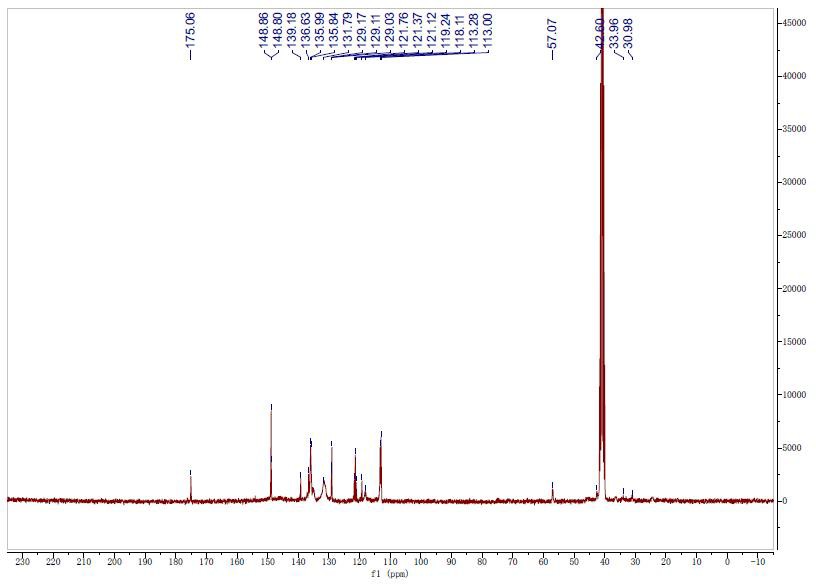


**Supplementary Figure 2.** ^13^C-NMR spectrum of compound **4a**


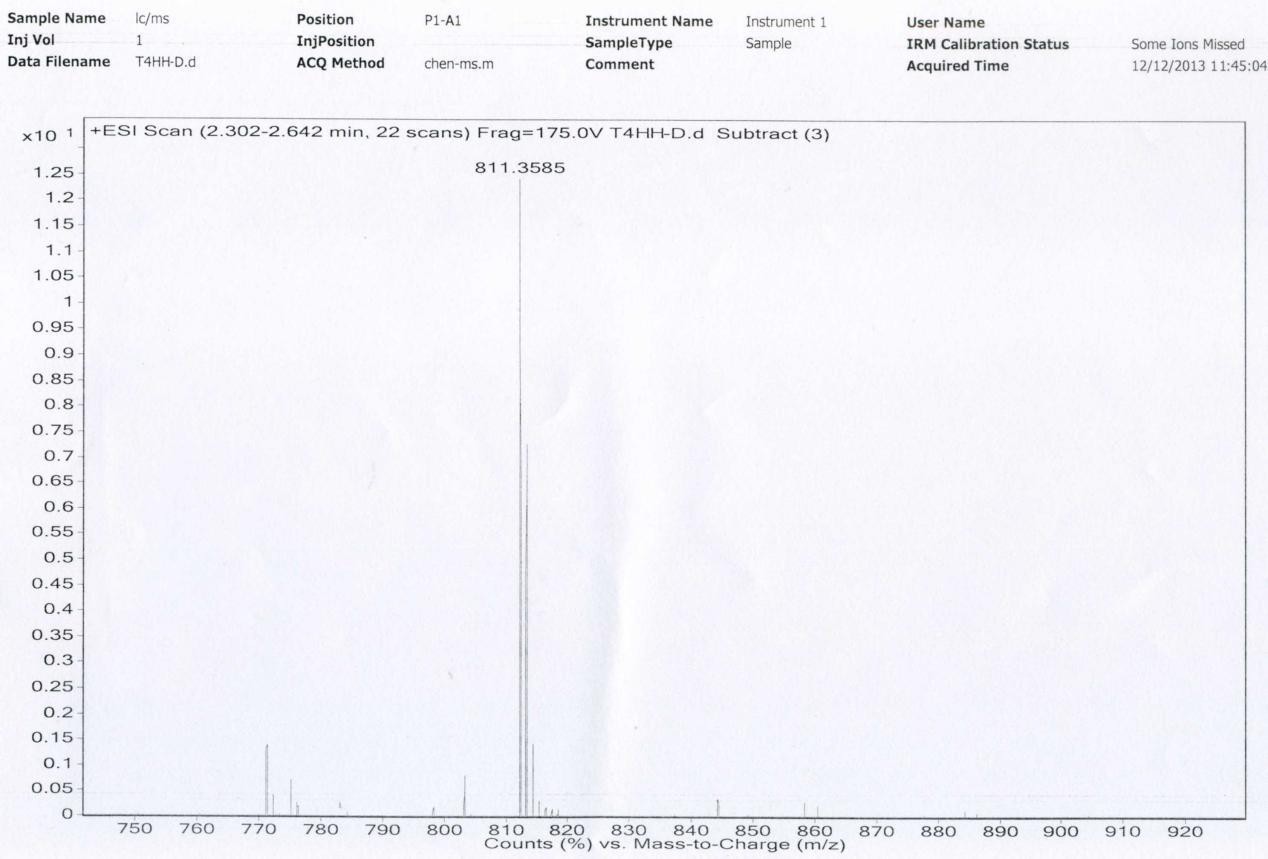


**Supplementary Figure 3.** HRMS spectrum of compound **4a**


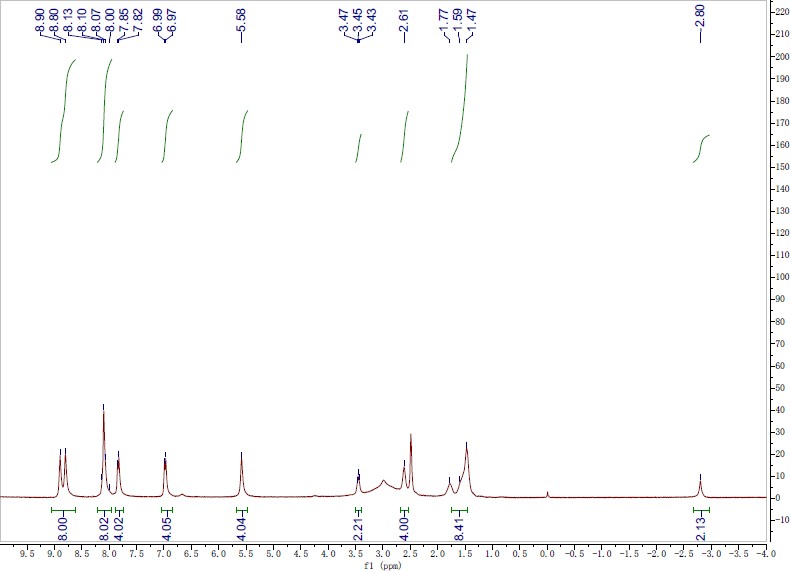


**Supplementary Figure 4.** ^1^H-NMR spectrum of compound **4b**

Supplementary Material


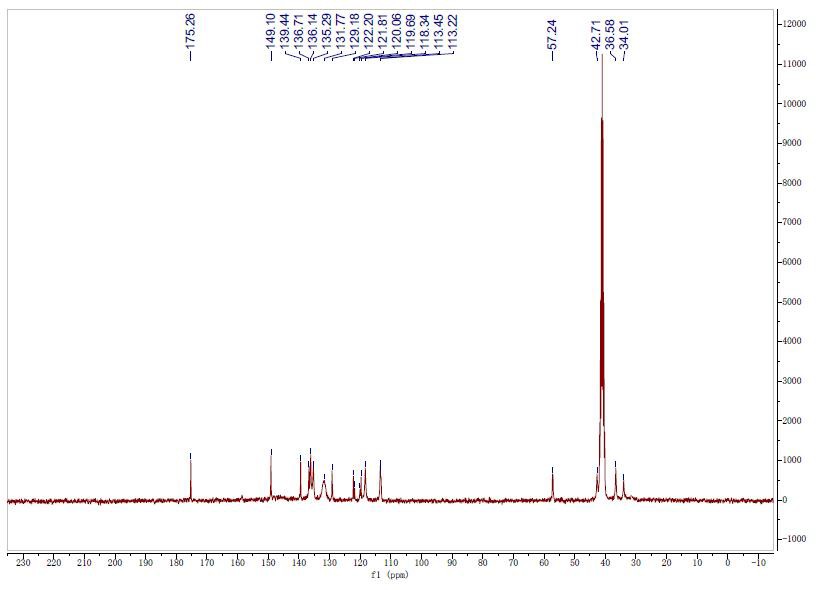


**Supplementary Figure 5.** ^13^C-NMR spectrum of compound **4b**


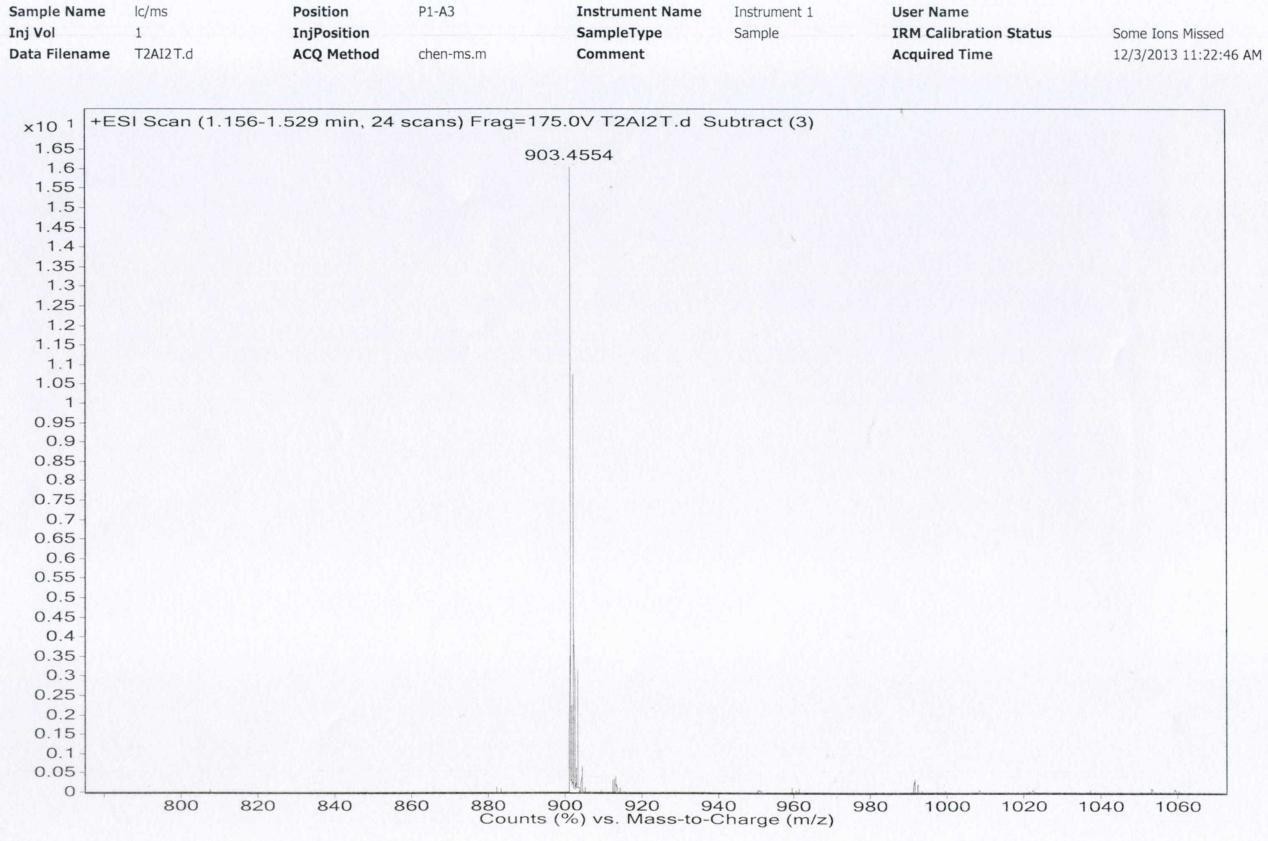


**Supplementary Figure 6.** HRMS spectrum of compound **4b**


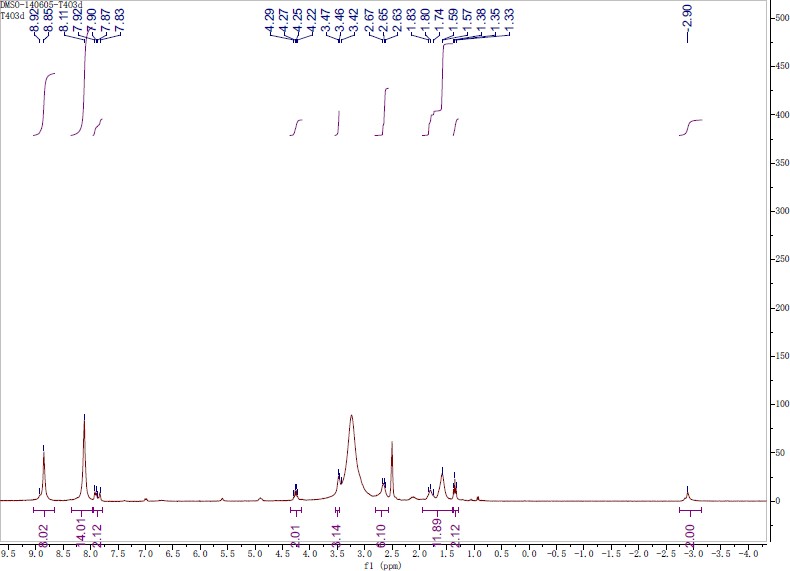


**Supplementary Figure 7.** ^1^H-NMR spectrum of compound **4c**


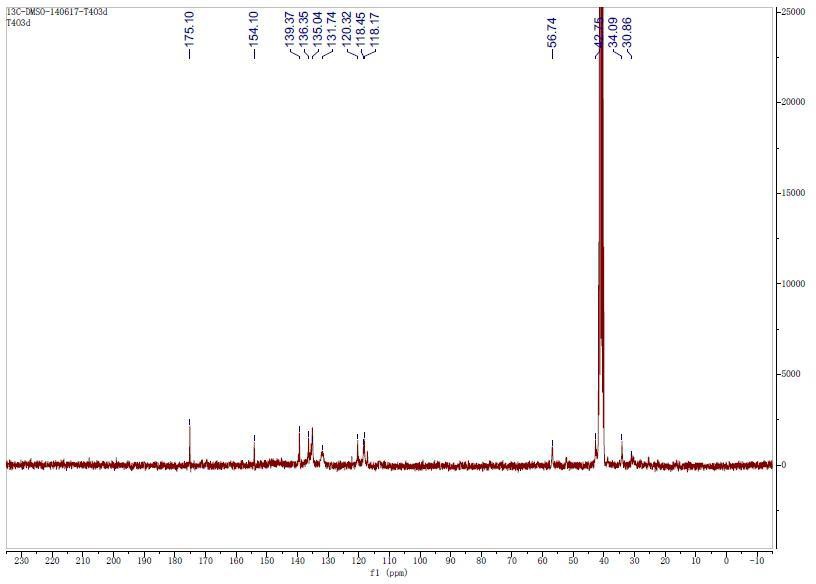


**Supplementary Figure 8.** ^13^C-NMR spectrum of compound **4c**

Supplementary Material


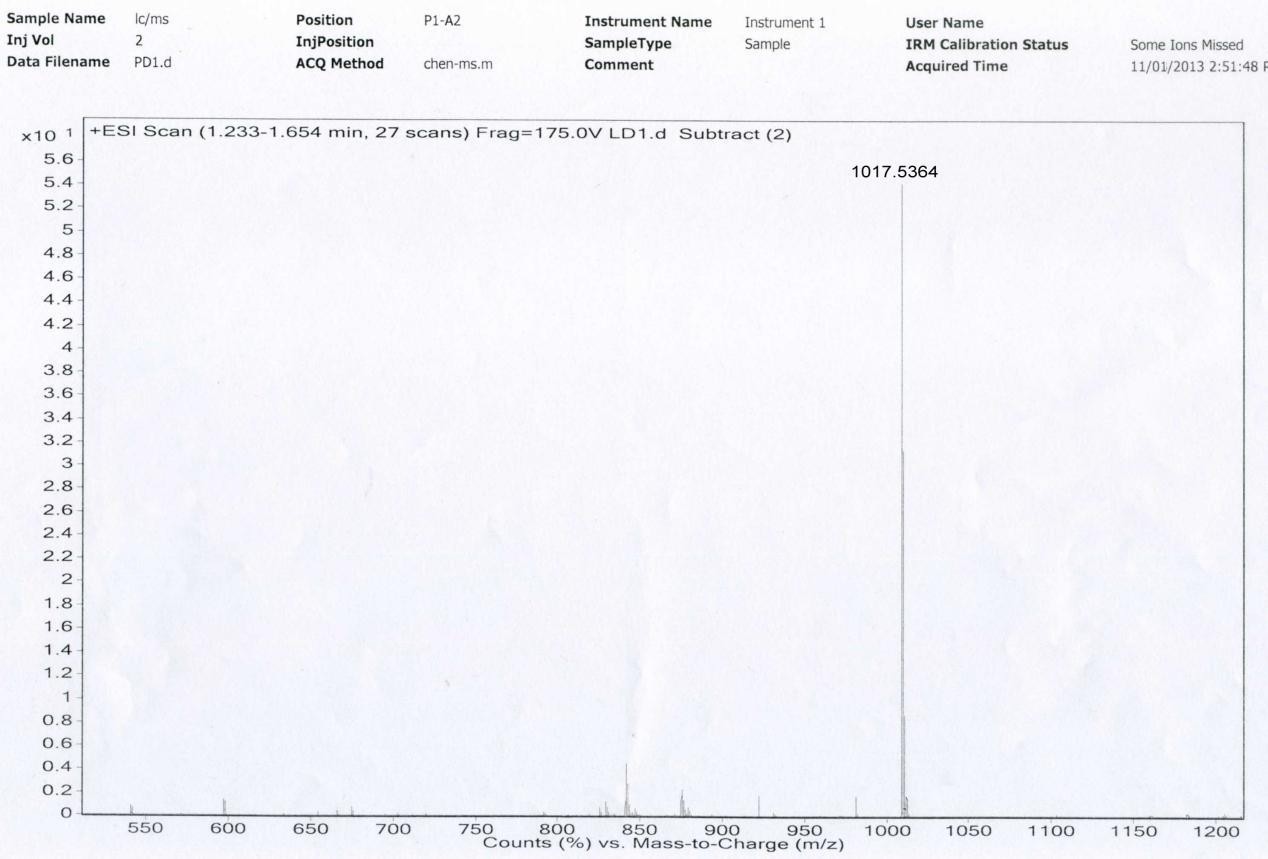


**Supplementary Figure 9.** HRMS spectrum of compound **4c**


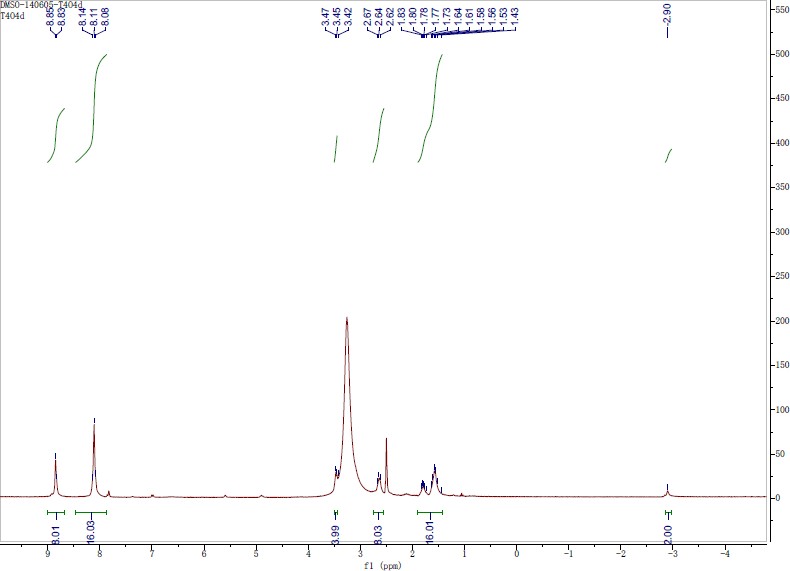


**Supplementary Figure 10.** ^1^H-NMR spectrum of compound **4d**


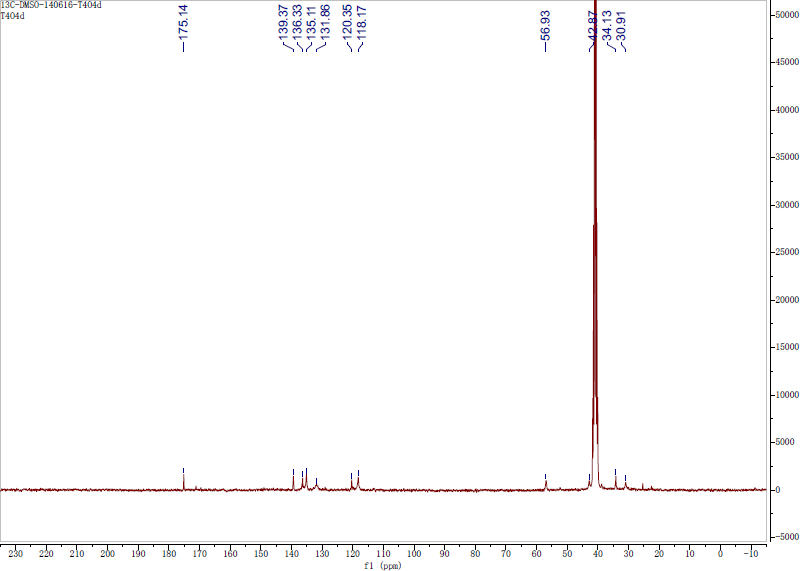


**Supplementary Figure 11.** ^13^C-NMR spectrum of compound **4d**


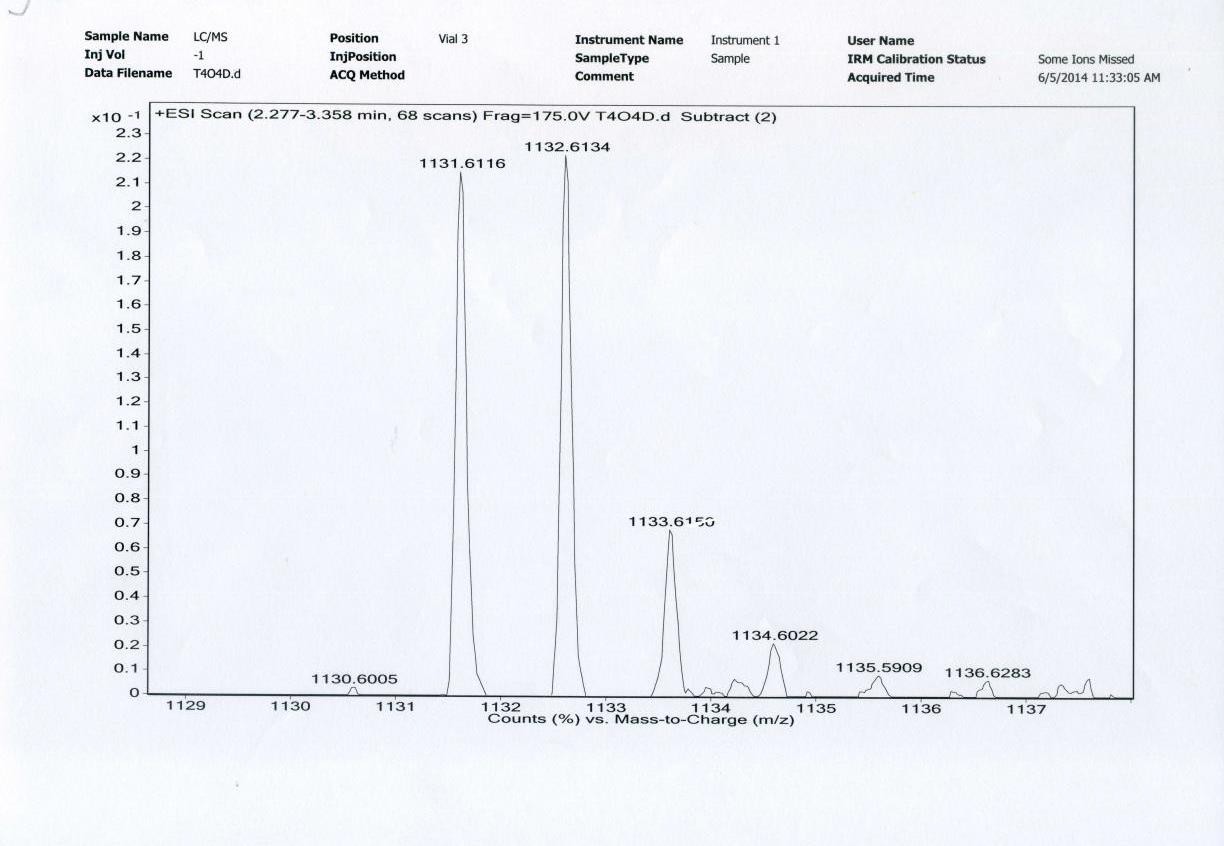


**Supplementary Figure 12.** HRMS spectrum of compound **4d**
